# Comparative codon usage analysis of HIV-1 and HIV-2 genomes

**DOI:** 10.1186/1471-2334-14-S3-E2

**Published:** 2014-05-27

**Authors:** KK Vidyavijayan, S Hassan, LK Precilla, S Swaminathan, LE Hanna

**Affiliations:** 1 Department of Clinical Research, National Institute for Research in Tuberculosis, Chennai, India

## Background

Compared to HIV-1, HIV-2 infection is associated with slower disease progression and transmission, longer latency period and low or undetectable plasmatic viral levels. In an attempt to understand underlying reasons for the varying pathogenicity between HIV-1 and HIV-2 at the molecular level, we analyzed the differences in codon usage pattern of all nine coding genes.

## Materials and methods

Thirty five full length HIV-2 genome sequences deposited during the period 1986-2008 were downloaded from LANL. The effective number of codons (NC), GC, GC3s and Relative Synonymous Codon Usage data corresponding to each gene were calculated using Codon W and Correspondence analysis (CA).

## Results

The NC for all the nine genes ranged from 43 – 55.2, indicating that the genes of HIV-2 are also poorly biased, as has been reported earlier for HIV-1, resulting in low levels of expression of these proteins in general. The NC value of the *tat* gene of HIV-2 was higher than that HIV-1. The CA of all nine genes revealed that the factors influencing the expression of different genes were not the same.

## Conclusion

The codon usage pattern of HIV-2 is distinctly different from that of HIV-1, and appears to be majorly shaped by the genomic composition rather than translational selection. The high Nc value of *tat* is suggestive of a lower level of expression of the protein in HIV-2 as compared to HIV-1, implying that this could be one of the viral factors responsible for lower levels of HIV-2 replication and delayed disease progression.

